# The Role of Estrogen Therapy as a Protective Factor for Alzheimer’s Disease and Dementia in Postmenopausal Women: A Comprehensive Review of the Literature

**DOI:** 10.7759/cureus.43053

**Published:** 2023-08-06

**Authors:** Noor Ali, Rohab Sohail, Syeda Rabab Jaffer, Sadia Siddique, Berfin Kaya, Inioluwa Atowoju, Alizay Imran, Whitney Wright, Spandana Pamulapati, Faiza Choudhry, Anum Akbar, Uzzam Ahmed Khawaja

**Affiliations:** 1 Obstetrics and Gynecology, Thumbay University Hospital, Ajman, ARE; 2 General Physician, Dubai Medical College, DXB, ARE; 3 Internal Medicine, Quaid-e-Azam Medical College, Bahawalpur, PAK; 4 Internal Medicine, Dow University of Health Sciences, Karachi, PAK; 5 Gastroenterology, Blackpool Victoria Hospital National Health Services (NHS) Foundation Trust, Blackpool, GBR; 6 Obstetrics and Gynaecology, Izmir Ataturk Research and Training Hospital, Izmir, TUR; 7 Obstetrics and Gynaecology, Izmir Kâtip Celebi University, Faculty of Medicine, Izmir, TUR; 8 Obstetrics and Gynecology, Kharkiv National Medical University, Kharkiv, UKR; 9 Surgery, Windsor University School of Medicine, Chicago, USA; 10 Obstetrics and Gynecology, Texila American University, Georgetown, GUY; 11 Obstetrics and Gynecology, Alluri Sita Rama Raju Academy of Medical Sciences, Eluru, IND; 12 Medicine and Surgery, Liaquat University of Medical and Health Sciences, Sindh, PAK; 13 Pediatrics, University of Nebraska Medical Center, Omaha, USA; 14 Pulmonary and Critical Care Medicine, Jinnah Medical and Dental College, Karachi, PAK; 15 Clinical and Translational Research, Dr Ferrer BioPharma, South Miami, USA

**Keywords:** post menopausal estrogen deficiency, estrogen therapy, estrogen receptor (er), alzheimers disease, dementia

## Abstract

The complete cessation of menstruation for 12 months with associated vasomotor symptoms is termed menopause. Apart from playing a role in reproduction, estrogen significantly affects the central nervous system (CNS). Population-based studies highlighted a substantial difference in the prevalence of dementia between men and women, with Alzheimer-associated dementia being more prevalent in women, indicating that estrogen deficiency might be a risk factor for neurodegenerative diseases. Patients with dementia experience a progressive decline in neurocognitive function, beginning with short-term memory loss that progresses to long-term memory loss and the inability to perform everyday activities, leading ultimately to death. There is currently no cure for dementia, so preventing or slowing the disease’s progression is paramount. Accordingly, researchers have widely studied the role of estrogen as a neuroprotective agent. Estrogen prevents dementia by augmenting Hippocampal and prefrontal cortex function, reducing neuroinflammation, preventing degradation of estrogen receptors, decreasing oxidative damage to the brain, and increasing cholinergic and serotonergic function. According to the window phase hypothesis, estrogen’s effect on preventing dementia is more pronounced if therapy is started early, during the first five years of menopause. Other studies like The Woman’s Health Initiative Memory Study (WHIMS) showed unfavorable effects of estrogen on the brain. This review aims to establish an understanding of the currently available data on estrogen’s effect on neurodegeneration, namely, dementia and Alzheimer’s disease.

## Introduction and background

Menopause is a physiologic phenomenon in females induced by estrogen deficiency. This clinical syndrome affects women at a median age of 51 years and mainly manifests as vasomotor symptoms. Given that multiple body parts are affected, including but not limited to the vagina, ovaries, bones, and arteries, other symptoms arise as well. This physiologic change starts in the ovaries. The number of ovarian follicles (and thus ovarian reserve) begins to decline, leading to reductions in inhibin and estradiol. Low estradiol levels perpetuate positive feedback to the hypothalamus. In response, the increase in gonadotrophin-releasing hormone (GnRH) production by the hypothalamus increases luteinizing hormone (LH) and follicle-stimulating hormone (FSH) production from the pituitary gland. These are the two main culprits for the vasomotor symptoms experienced in menopause. Because Estrogen is the main driver for endometrial proliferation, menstruation initially becomes irregular, ultimately ceasing altogether [1]. The role of estrogen surpasses the ovaries. This is proven by the myriad of symptoms experienced by post-menopausal women apart from the vasomotor ones. Estrogen helps maintain a woman's urogenital tract; hence, loss of vulvar fullness, narrowing of the introitus, and mucosal inflammation develop in menopause. As a result, women experience vaginal dryness, vaginal irritation, painful intercourse, incontinence, and increased urinary tract infections (UTIs) [2]. Estrogen also plays a role in bone growth and remodeling. It stimulates bone formation by activating osteoclasts and osteoblasts. Lower estrogen levels increase bone resorption compared to bone formation [3]. This imbalance in bone remodeling puts menopausal women at risk for osteopenia, which, if not managed, leads to osteoporosis subsequently, and an increased risk for fractures [4,5]. Another vital function of estrogen is preventing cardiovascular disease by optimizing lipid metabolism and blood vessel resistance. Lack of estrogen negatively alters the endothelium of the tunica intima, leading to vasoconstriction and possible hypertension. An accelerated rise in low-density lipoprotein (LDL) levels is also seen in menopause, increasing the risk of adverse cardiovascular events [1,4]. It is estimated that women spend more than a third of their lifetime in the postmenopausal period, and given that the effects of menopause are vast and can significantly affect a woman's quality of life (QOL), there have been countless studies and research attempts at determining preventive methods and developing treatment modalities to combat postmenopausal diseases and enhance QOL. However, definitive results are yet to be seen. There are several treatments for symptom control and disease prevention. These include lifestyle modifications (e.g., dietary changes), non-hormonal (e.g., selective reuptake inhibitors), and hormonal therapies. The FDA has approved hormone replacement therapy (HRT) for treating vasomotor symptoms, i.e., hot flashes, night sweats, and vaginal dryness. HRT decreases the risk of osteoporosis in menopausal women [6-8]. Despite the positive effects of estrogen therapy, studies show that outcomes vary tremendously depending on a myriad of factors, like age, race, smoking status, and history of breast cancer, to name a few. Health professionals should thoroughly evaluate the benefits and risks of estrogen therapy for each woman before prescribing it [9].

## Review

Estrogen therapy and its effects on different organ systems 

Estrogen Therapy and Cardiovascular System

The principal cause of death in postmenopausal women is coronary heart disease (CHD). It is estimated that about 3 to 5 cases per 1000 are reported in low-risk women annually [9]. In 1996, WHO released epidemiological findings proposing a 44% reduction in the risk of death by CHD in HRT users compared to non-users. However, a more recent survey, the Nurses’ Health Study, showed a 39% reduction in the risk of death by CHD in women using HRT when considering the participant's pre-existing cardiovascular risk factors. The Woman's Health Initiative in 1998 demonstrated the cardioprotective role of estrogen therapy. However, a higher risk of developing venous thromboembolism (VTE) was observed in women taking continuous estrogen therapy (ET) compared to women taking a placebo. About five in 1000 women taking ET developed VTE, compared to two in 1000 women taking placebo. This risk was highest during the first two years and declined at the seven-year follow-up mark. At the same time, the Heart and Estrogen/Progestin Replacement Study (HERS) in 1998 showed no significant difference in the VTE risk between users and non-users during the follow-up period. Hence, at this time, it is best to avoid using ET as a prevention or treatment for CHD, given the constant risk of VTE. The current sensible approach to curbing CHD in postmenopausal women is to encourage the maintenance of a healthy BMI and follow a heart-healthy diet [10,11].

Estrogen Therapy and Skeletal System

The risk of osteopenia and osteoporosis increases after menopause. Accordingly, bone fractures are more prevalent in older women than in older men, with the highest prevalence seen in white women and the lowest prevalence in black women. In 1998, WHIMS observed a significant decrease in the risk of postmenopausal hip fractures after ET, even after a 13-year follow-up period. Time and time again, studies have replicated the efficacy of ET in preventing postmenopausal osteoporosis. Notably, these effects were detected in women <60 years of age who have been menopausal for <10 years [12-14].

Estrogen Therapy and the Urogenital System

As estrogen receptors are found in the urethral mucosa and muscles, urinary incontinence in postmenopausal women can be managed with ET. Furthermore, administering ET directly in the vagina, in the form of creams or tablets, is more effective in relieving symptoms than oral or parenteral routes since these forms release estrogen onto the vaginal tissue [9]. A randomized controlled study involving 88 postmenopausal women concluded that intravaginal estriol improved urinary incontinence symptoms and may be used as an alternative to HRT. On the other hand, HERS observed worsening symptoms in incontinent women at baseline [15,16].

Cognitive Decline

Dementia is a progressive neurodegenerative disease affecting memory, problem-solving skills, language, and cognitive thinking, ultimately impairing everyday activities. Dementia prevention relies on interventions that control and modify risk factors. The disabling nature of dementia and its monumental emotional, psychological, and social ramifications make studying it a crucial and worthwhile endeavor. Especially given that the prevalence of dementia increases from 1.5% in the sixth decade of life to 40% in the ninth decade of life. In 2010, it was reported that 35.6 million people were living with dementia [17]. There are several forms of dementia, such as Alzheimer’s disease-associated dementia, vascular dementia, Pick’s disease-induced frontotemporal dementia, and Lewy body dementia. The incidence of Alzheimer’s disease (AD), the most prevalent cause of dementia, doubles every fifth year in people aged >65 years [18-20]. AD is rapidly becoming a pertinent public health problem in the United States. Recent studies indicate that more than four million Americans are affected by AD. The annual cost of caring for these patients is estimated to be over $100 billion. By 2050, at least 14 million Americans will suffer from this disease [21].

Many epidemiologic studies postulate that the decline in estrogen levels accounts for the increased risk of AD in postmenopausal women. Additionally, faster cognitive decline has been noted in women with early-onset menopause. Likewise, a multitude of observational data supports the use of HRT in reducing the risk of dementia, primarily AD. Other reputable clinical studies proved a higher lifelong risk of dementia and cognitive impairment in patients who underwent bilateral or unilateral oophorectomy before their natural onset of menopause. This risk was diminished by initiating ET after the operation [17,22-24]. However, these findings are challenged by the Women Health Initiative (WHI) trial, the most comprehensive study on the cognitive consequences of hormonal therapy [22-24]. This study suggests an increased risk of AD in postmenopausal women over 65 who are prescribed oral estrogens, with or without progestin. The study ceased prematurely due to the increased risk of CVD and other complications associated with using estrogen-based hormone therapy, and follow-ups of this study have been underpowered [23].

**Figure 1:**
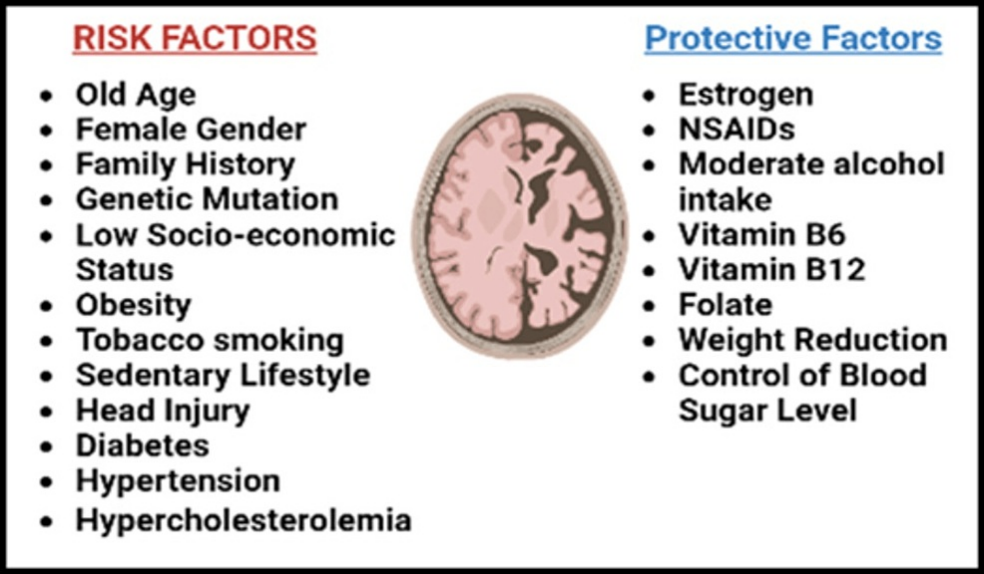
Precipitating and protective factors associated with Alzheimer’s disease. Created with bioRender.com

Early-onset Alzheimer's is seen in patients with genetic mutations, such as Down’s syndrome and the apolipoprotein-epsilon allele, predisposing them to develop this neurological disorder [18,19,24,25]. In addition, studies have found several modifiable and non-modifiable factors associated with disease development, such as gender (more common in females), family history, lifestyle (cigarette smoking, sedentary lifestyle), head injury, low socioeconomic status, and diseases like diabetes, hypertension, hypercholesterolemia, obesity, cardiovascular diseases, etc. [18,25-28]. Generally, dementia is more common in men than women, but the prevalence of Alzheimer-associated dementia in post-menopausal women is found to be higher; this has led to the discovery of low estrogen levels as a risk factor for Alzheimer’s disease [9,24]. Estrogen therapy in post-menopausal women, NSAIDs for treatment of joint pain, low-dose alcohol intake, weight reduction, reasonable control of diabetes and hypertension, and a diet rich in vitamin B6, B12, and folate are associated with protection against the development of Alzheimer [19,21,28,29]. Figure 1 summarizes multiple risk and protective factors associated with Alzheimer's.

The first symptom of Alzheimer's is memory impairment, primarily affecting short-term memory [9,18,30]. The Montreal Cognitive Assessment Scale detects cognitive defects [26]. Patients later develop word-finding difficulty and an inability to perform executive tasks [21]. As the disease progresses, the patients exhibit behavioral symptoms like depression, psychosis, hallucinations, or illusions, spatial disorientation (getting lost in familiar neighborhoods), language problems, inability to perform everyday tasks, making the patient dependent, and ultimately, death [18,21,24,28]. Investigations show diffuse cortical atrophy predominantly involving the temporal lobe and hippocampus (an area of the brain involved in memory) and ventriculomegaly. In addition, on biopsy, neurofibrillary tangles and senile plaques are seen [8,18,26,30-32].

**Figure 2:**
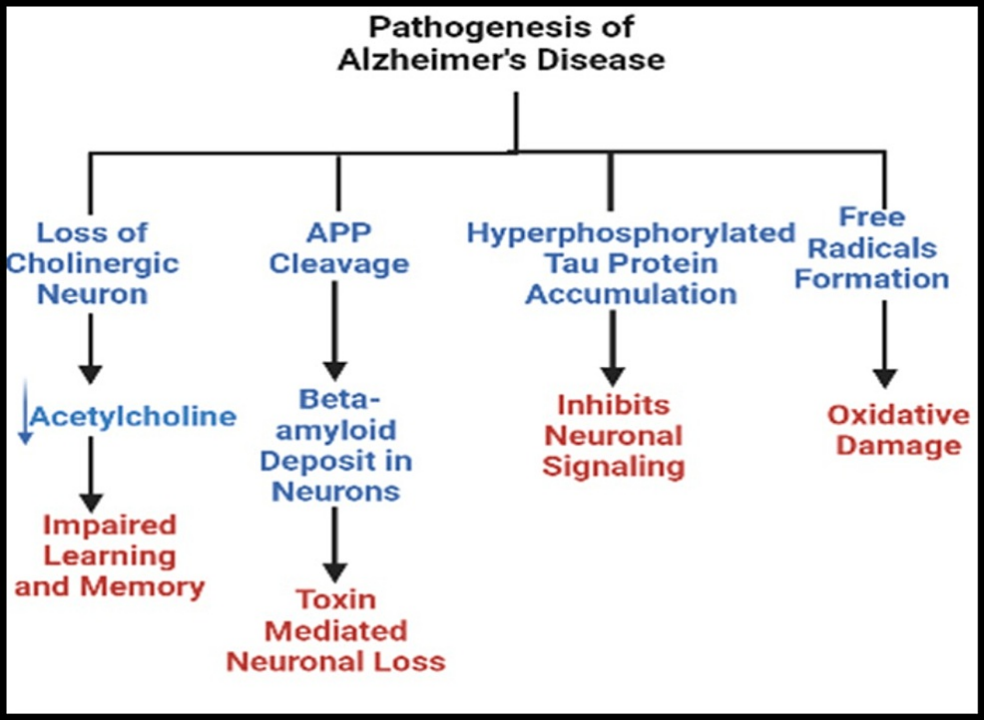
Pathogenesis of Alzheimer’s disease. Created with bioRender.com

The pathogenesis of Alzheimer’s disease begins with the breakdown of the amyloid precursor proteins to produce beta-amyloid, which accumulates around the cerebral blood vessels, forming amyloid plaques [8,26,32-34]. Amyloid plaques weaken the vessel walls, leading to intracranial hemorrhage. Besides this, beta-amyloid also has a neurotoxic effect on the brain, destroying neurons and causing atrophy of the cortex [23,30,35,36]. In addition, beta-amyloid accumulation in the nucleus of the meynert (forebrain) causes the loss of acetylcholine, the neurotransmitter essential for memory and learning [28,29,32]. The tau protein (a naturally occurring protein bound to microtubules involved in neuronal transmission) detaches from microtubules and forms clumps inside neurons, inhibiting signal transduction [23,35,37]. Free radical-mediated oxidative damage is also involved in the development of neurodegeneration [28,30,34,38]. Figure 2 provides a summary of all the mechanisms involved in developing Alzheimer’s disease.

With the improvement in life expectancy, the prevalence of Alzheimer's has dramatically increased [18,32]. Yet the currently available treatment options neither improve nor slow the disease’s progression [32]. As Alzheimer's is the only cause of dementia more common in females than males, scientists focus on the relationship between female gonadal hormones and Alzheimer’s disease [18,21,24,37]. However, ambiguity exists regarding the extent of estrogen's involvement in menopause-associated cognitive decline and whether HRT can be used to prevent AD [39].

Estrogen

Estrogen, a steroid hormone, is synthesized in both genders. In females, it is involved in the development of the reproductive system and contributes to both primary and secondary sexual characteristics. Evidence shows that it exerts both neuroprotective and neuropathological effects, making it a bit complex to study. Interestingly, studies on the use of HRT in postmenopausal women suggest a possible protective effect against cognitive decline, reporting maintenance of brain tissue integrity with HRT [25]. They suggest that supplemental estrogen protects cognition by promoting cholinergic activity, protecting from toxic insult, stimulating neuron synthesis, and reducing deposition and enhancing the clearance of β-amyloid (the main constituent of the amyloid plaques in Alzheimer’s disease) [33,40]. On the other hand, the prothrombotic effect of estrogen may counteract its neurovascular preservative effect, given that it increases inflammatory markers and stroke risk, both of which are associated with the development of dementia [24,41]. The effects of HRT exposure have been thought to be influenced by various factors, like the duration of endogenous estrogen exposure, the type of estrogen formulation, and the concurrent use of progesterone [36].

Serum 17-beta estradiol levels are used to assess estrogen therapy’s effects on Alzheimer’s disease and the pathogenesis of dementia [22]. The estrogen used in ET acts on the peripheral estrogen receptor alpha and thereby causes a variety of adverse effects. A few studies assessing non-feminizing estrogens found that the most effective forms contain the two and four carbons of the phenolic ring of estrogen. These forms do not bind to the estrogen receptor but exhibit the neuroprotective actions of feminizing estrogens [42]. Non-feminizing estrogens were also found to be 8-114 times more potent than 17-beta estradiol. Therefore, these studies suggest using non-feminizing estrogens in ET in postmenopausal women. In the section ahead, we delve more into estrogen and its receptors and the multiple mechanisms of action proposed for their role in Alzheimer’s disease.

Estrogen Receptors 

There are three types of estrogen receptors (ER) in the body. Two are nuclear receptors, ERα and ERβ. The third is a membrane-bound G-protein-coupled receptor. Nuclear receptors are present in the CVS and CNS. They have transcriptional activities and participate in complex pathways pertaining to normal physiology. ERα is specifically present in the bone, mammary glands, prostate, liver, adipose tissues, uterus, and ovaries (theca cells). While ERβ is found in the colon, bladder, immune system, adipose tissues, and ovaries (granulosa cells). The membrane-bound G-protein-coupled receptor lacks transcriptional activity and is instead involved in non-genomic actions. It can cross-react with the other two nuclear ERs.

Transcriptional Activity of Nuclear Estrogen Receptors

Nuclear ERs undergo conformational changes upon activation by estrogen. Estrogen causes their dimerization, allowing them to move into the nucleus and bind to specific DNA sequences that regulate the expression of estrogen-responsive genes. The functions of nuclear ERs differ depending on their expression in the body. Some examples are maintaining cell structure, cell proliferation, and blood pressure regulation.

Splice Variants

ERs have many variants, and hence participate in different organ functions, affecting physiological and pathological processes. With age, the gene expression of spliced ERα mRNAs and aromatase enzymes declines, especially in brain areas concerned with memory and learning. This change is also seen in AD, suggesting estrogen’s role in the pathophysiology of AD [34].

Mitochondrial Dysfunction

**Figure 3:**
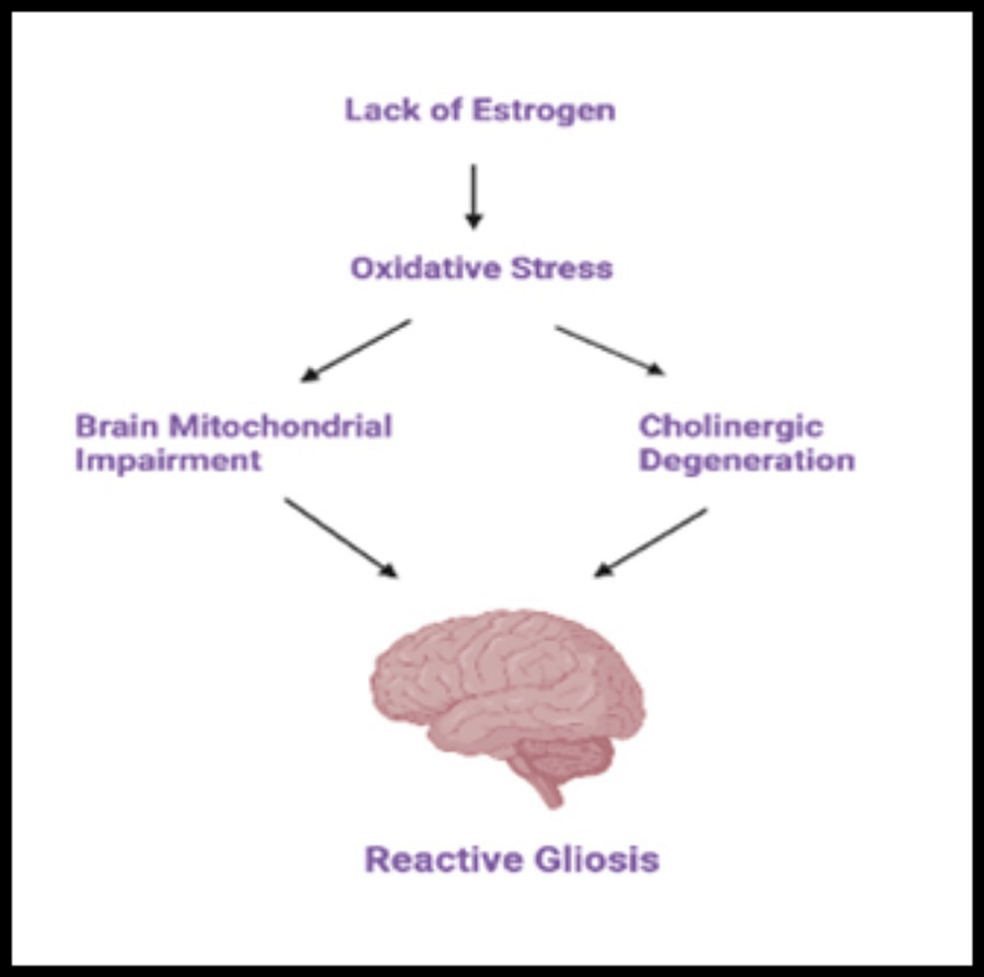
Mitochondrial dysfunction during menopause. Created with bioRender.com

Brain mitochondria are involved in brain energy production, oxidative stress regulation, and apoptosis. Oxidative stress accelerates with aging, accumulating reactive oxygen species (ROS) that damage lipids, proteins, and mitochondrial DNA. Mitochondrial dysfunction from oxidative stress is the first pathologic change observed in the aging brain and many neurodegenerative diseases, including AD. Proteomic studies highlight the role of estrogen in the expression of mitochondria. Long-term estrogen deficiency in postmenopausal women leads not only to altered ER expression but also to exacerbated oxidative stress in the brain, giving rise to mitochondrial impairment and cholinergic degeneration, ending eventually in reactive gliosis, as shown below in Figure 3. Correspondingly, brain mitochondria are believed to be the targets for estrogen’s neuroprotective effect [43].

Estrogen and the Cholinergic System

Neuroendocrine studies show that the cholinergic system plays an important role in memory and learning. This is particularly true for the septo-hippocampal cholinergic pathway. The cholinergic system, i.e., acetylcholine and choline-acetyltransferase activity, declines in AD, and long-term ET trials in postmenopausal women show improved cholinergic functions. Moreover, similar trials observed enhanced responsiveness to dopaminergic and serotonergic neurotransmitters. Granted, these studies have limitations; they do not directly analyze the effect of estrogen on receptor response at the hypothalamic pituitary axis. Plus, they were observational and cross-sectional [44]. Evidence from neuroimaging like PET and functional MRI scans demonstrates memory augmentation by estrogen via changes in the hippocampus and prefrontal cortex [45]. The Baltimore longitudinal study on aging observed the same. Its follow-up study examined the effects of hormone therapy over three years and reported increased blood flow to many areas of the brain, including the hippocampus, right posterior hippocampal cortex, and right entorhinal cortex, in women taking HRT compared to women who did not [45]. Considering the above, a few studies suggest using cholinergic-enhancing drugs in combination with ET to treat older postmenopausal women with early signs of mild cognitive impairment.

Neuroinflammation in Alzheimer's Disease

**Figure 4:**
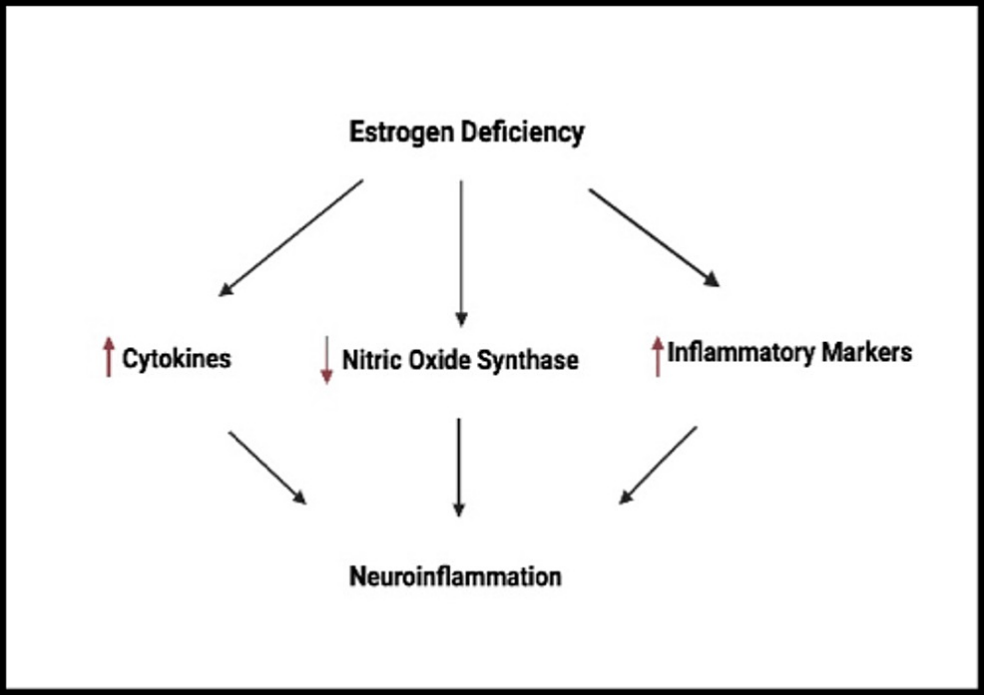
Cellular effects of estrogen deficiency. Created with bioRender.com

Neuroinflammation is the major mechanism involved in the pathogenesis of AD. Estrogen is reported to reduce inflammation by decreasing the levels of pro-inflammatory cytokines. Estrogen also stimulates the microglia to produce anti-inflammatory cytokines. One trial reported that estrogen treatment in female mice with autoimmune encephalomyelitis decreased the expression of proinflammatory cytokines, TNFα, IFN-γ, and chemokines. Female ovariectomized mice with lower baseline estrogen levels showed increased expression of these cytokines. Therefore, a lack of estrogen can lead to increased cytokines and inflammatory markers. In addition, ERβ agonists seem to affect the levels of interleukins, like IL-6, IL-1β, and ΙL23-p19, and produce nitric oxide synthase (NOS), which reduces the astrocytes and microglia-mediated inflammatory response. While ERα agonists downregulate chemokines, microglial activation, astrogliosis, and T-cell infiltration in mice, hence the neuroprotective effects of estrogen [34]. Figure 4 depicts the cellular response to estrogen deficiency.

Ovariectomized mice that were given Aβ infusion depicted an increment in microgliosis, astrogliosis, and nuclear factor kappa-B (NF-ĸB) activation. Furthermore, in the human brain, estrogen administration increased Aβ clearance by microglial cells, emphasizing estrogen’s role in microglia-mediated Aβ clearance leading to the downregulation of inflammation [34].

*Alzheimer's Disease and Genetic Polymorphism in the APOE4* *Gene*

The APOE genotype is strongly associated with the risk of AD and cognitive decline. Compared to the wild-type APOE3/E3, those with APOE3/E4 and APOE4/E4 are at a 3-4-fold and 12-15-fold increased risk of AD, respectively [46]. APOE4 is expressed in more than half of the patients diagnosed with AD. Interestingly, APOE2 is proven to be neuroprotective. In humans, APOE4 has three isoforms: APOE2, APOE3, and APOE4 with a prevalence of approximately 10%, 80%, and 14%, respectively. Apo E is found in the brain and functions as a lipid transporter in CSF. Estrogen is involved in cholesterol and lipid transport and modulates low-density lipoprotein receptor-related protein, which plays a role in Aβ production and clearance. The bond between lipoprotein receptor-related protein 1 and APOE4 is weak, impairing Aβ clearance and leading to neuroinflammation in AD. Many studies have shown the above-mentioned relationship between APOE4-carrier women and the development of AD in relation to hormone replacement therapy (HRT). The use of HRT in postmenopausal women with the APOE4 mutation decreases Aβ deposition [34]. Saleh et al. conducted a study revealing the influence of the APOE genotype and the age at which HRT is initiated on cognitive function, brain volumes related to cognition, and their varying outcomes. The findings indicate that APOE4 women are most responsive to HRT. They exhibited larger entorhinal cortex and amygdala volumes, along with higher repeatable battery for the assessment of neuropsychological status (RBANS) delayed memory index scores compared to APOE4-non-HRT users. Additionally, an earlier age of HRT initiation was associated with a larger hippocampus volume in APOE4 women. Earlier studies reported higher cerebral blood flow (CBF) in APOE4 women. Thus, HRT’s response can be described in an APOE-dependent manner [46].

The window of opportunity theory 

Multiple studies indicate an optimal period, or rather, a ‘Window of Opportunity’ for prescribing hormone therapy that results in better cognitive outcomes in post-menopausal women [25,36,41,47,48]. This period is hypothesized to be during perimenopause or right after menopause, specifically during the first five years [48]. A couple of reasons could explain this, based on various observational and interventional clinical studies. First, a long premenopausal phase is associated with greater cognitive function and a decreased risk of AD. The risk of AD is reduced by 0.5% for every additional month of estrogen exposure before menopause [49]. Second, there is a reduction in the number and affinity of ERs, especially ERα, to estrogen with the prolongation of the hypoestrogenic phase [31,49,50]. Patients with AD exhibit a straight-line relationship between the number of ERα and cognitive function, which further supports the idea of ‘ERα degradation’ and a less-than-optimal response to hormone therapy, no response, or even negative outcomes when initiated after a certain period [49]. Such unfavorable results were seen in the Women's Health Initiative Memory Study (WHIMS), which found that the risk of cognitive impairment was significantly increased in postmenopausal women aged 65 or above taking continuous combined hormone therapy, and in the WHIMS-MRI study that revealed older women taking hormone therapy have more substantial brain atrophy than their non-user counterparts [50,51]. Likewise, a study conducted by Whitmer et al. demonstrated a reduced (26%) risk of dementia when initiating HRT at the age of 48.7 years, compared to an increased (48%) risk when initiating it at the age of 76 years [52]. Additionally, a 2016 study evaluating structural brain changes associated with hormone therapy using optimally discriminative voxel-based analysis (ODVBA) resulted in unfavorable gray matter changes in those receiving hormone therapy [53]. It’s been postulated that these adverse effects of ET could be due to lower baseline cognition or pre-existing degenerative pathology affecting the nervous system [51].

It is worth mentioning that most studies associating ET with a reduced risk of AD were done on younger females. Third, given endogenous estrogen’s positive and protective neurocognitive roles, there would be less neural damage during the initial years of menopause compared to later years, and thus a greater potential for delaying or preventing mild cognitive impairment and, ultimately, AD [23,36]. An example of this is a 2016 randomized hormone therapy trial that compared recent postmenopausal women receiving transdermal 17β-estradiol with those receiving placebo; the results showed less Aβ deposition in those receiving hormone therapy compared to placebo [48]. These findings support estrogen’s antioxidant activity observed in lab mice injected with Aβ42 [54]. Another example is a study that looked at the effects of estrogen therapy on postmenopausal women with reduced CBF. By the end of week six, cerebral blood flow was back to normal [55]. Accordingly, women exposed to extended hypoestrogenic phases would likely suffer irreversible brain damage leading to cognitive defects due to the prolonged cerebral blood flow compromise. At that point, estrogen would not be beneficial [31].

Unlike the apparent consensus on the plausibility of the ‘critical window’ hypothesis, there are mixed findings on the duration of hormone therapy that is the safest and most efficacious in preventing or treating AD. Laboratory animal studies demonstrate neurogenesis with short-term estrogen therapy, whereas long term estrogen exposure halts it. Similarly, a 2009 randomized control trial studying the effect of a 4-6-year hormone therapy course on the brain MRI profiles of older women revealed reduced hippocampal sizes. They concluded that a short estrogen course might yield better cognitive effects [31]. However, results from a systematic review published in 2021 suggested protection against AD with hormone therapy use beyond 10 years, irrespective of the initiation time [48]. Furthermore, a recent 2023 cohort study found that AD was present in most (88%) women with limbic-predominant age-related TDP3 encephalopathy neuropathologic change (LATE-NC). This is significant given that they discovered that long-term estrogen therapy in the 50s and 60s reduced the chances of limbic-predominant age-related TDP3 encephalopathy neuropathologic change (LATE-NC) being discovered at death [56]. Moving on to estrogen therapy formulations, we note that there are two main forms, unopposed estrogen, i.e., conjugated equine estrogens (CEE), and opposed estrogen, i.e., estrogen plus medroxyprogesterone (CEE + MPA). The progesterone component is added for women with an intact uterus to protect against endometrial hyperplasia. Differentiating between the two formulations is important, given their different effects on dementia [41]. In the WHIMS trial, CEE + MPA’s effect on cognition was compared against that of the placebo; results showed an increased risk for dementia with CEE + MPA [23]. This suggests that progesterone blocks beneficial neural estrogen pathways or perpetuates estrogen’s neurotoxicity.

Another randomized trial displayed similar undesirable effects for progesterone when looking at metabolic brain changes with different hormone therapy formulations. Continuing estrogen therapy, whether 17βE or CEE-based, with progesterone resulted in noticeable deterioration in the posterior cingulate/precuneus area. Metabolic changes in this area have been linked to cognitive impairment in AD. The usage of unopposed 17βE appears to have promising results, mediated by its ability to delay the onset of cognitive impairment, as women who continued using unopposed 17βE had preserved metabolism of the posterior cingulate cortex [25].

One thing is for sure, given the multiple studies displaying mostly negative effects or no effect of starting hormone therapy at an older age, it is currently not recommended to prescribe estrogen therapy to women >65 years of age for the prevention or treatment of AD, especially if they already suffer from some degree of cognitive impairment [23,36,52,57]. Until more thorough research is conducted or safer treatment methods are generated, it is best to assess the patient’s educational status and medical history, including genetic history, family history, and cardiovascular health. As well as considering their age, treatment timing, dose, and duration before recommending HRT [58].

## Conclusions

On average, a woman’s lifespan is longer than that of a man’s, and a significant part of that life is spent in the post-menopausal state. Hence, women’s quality of life is threatened by the myriad of negative changes resulting from the lack of estrogen. Cognitive decline is a major one. Posing a time-sensitive challenge for primary care and women health providers. Despite the promising positive cognitive effects estrogen therapy has displayed in laboratory studies, this has yet to be strongly and unequivocally replicated in human studies. Thus far, estrogen therapy prescribed alone, i.e., without progesterone, solely for preventing Alzheimer’s disease and dementia, appears to best benefit healthy, cognitively intact peri-menopausal women under the age of 65 years. Given the scarcity of studies looking at the most effective duration for HRT and the known pro-thrombotic effects of estrogen, the length of therapy would be up to the physician’s discretion, guided by his or her initial and serial assessments of the patient. Based on the literature reviewed, estrogen is mostly protective, not curative. To solidify this and be able to incorporate estrogen therapy in the preventive management of dementia in women, we need larger-scale studies specifically targeting women with risk factors for cognitive decline, like women with a family history of dementia or early-onset dementia, APOE4 carriers, women with early-onset menopause, such as those with premature ovarian insufficiency, and women with cerebrovascular disease. Furthermore, we noted estrogen’s involvement in neuroprotective signaling pathways. All signaling pathways require enzymes and/or co-factors, and those require vitamins. Accordingly, correcting vitamin deficiencies before studying the effects of estrogen on cognition is pivotal.
